# Use of NSAIDs, smoking and lung cancer risk

**DOI:** 10.1038/sj.bjc.6604151

**Published:** 2007-12-18

**Authors:** J H Olsen, S Friis, A H Poulsen, J Fryzek, H Harving, A Tjønneland, H T Sørensen, W Blot

**Affiliations:** 1 Department of Genetics and Medical Treatment, Institute of Cancer Epidemiology, Danish Cancer Society, Copenhagen, Denmark; 2 International Epidemiology Institute, Rockville, MD, USA; 3 Pulmonary Unit, Aalborg Hospital, Aalborg, Denmark; 4 Department of Clinical Epidemiology, Aarhus University Hospital, Aarhus, Denmark; 5 Department of Medicine, Vanderbilt University Medical Center, Vanderbilt-Ingram Comprehensive Cancer Center, Nashville, TN, USA

**Keywords:** lung cancer, pharmacoepidemiology, non-steroidal anti-inflammatory drugs, chemoprevention

## Abstract

We investigated the risk of lung cancer in relation to non-steroidal anti-inflammatory drugs (NSAIDs) among 573 cases and 857 sex- and age-matched controls for whom we had information on use of NSAIDs, from a prescription database covering all pharmacies in Denmark since 1995, and self-reported NSAID use, smoking habits and other potential confounders. Associations were expressed as odds ratios, assessed by logistic regression in unmatched analyses. After controlling for smoking habits, length of education and concomitant use of acetaminophen, we found a slightly decreased relative risk of 0.86 (95% confidence intervals, 0.65–1.14) for lung cancer associated with any use of NSAIDs. The risk decreased significantly (*P*=0.02) with increasing numbers of dispensed prescriptions per year during the 1–3 years before the index date with a relative risk of 0.49 (0.28–0.84) among those with four or more prescriptions per year during this period. Our findings suggest that regular use of NSAIDs is associated with a slightly or moderately reduced risk for lung cancer.

Aspirin and other non-steroidal anti-inflammatory drugs (NSAIDs) used in relation to lung cancer have been investigated in at least 20 studies, including four hospital-based case–control studies (Rosenberg, 1995; Harris *et al*, 2002; Moysich *et al*, 2002; Muscat *et al*, 2003), fourteen cohort studies (Paganini-Hill *et al*, 1989; Thun *et al*, 1993; Schreinemachers and Everson, 1994; Langman *et al*, 2000; Akhmedkhanov *et al*, 2002; Friis *et al*, 2003; Holick *et al*, 2003; Sørensen *et al*, 2003; Ratnasinghe *et al*, 2004; Skriver *et al*, 2005; Hayes *et al*, 2006; Hernández-Diaz and Rodriguez, 2007; Jacobs *et al*, 2007; Wall *et al*, 2007) and three randomised trials (Peto *et al*, 1988; Lee *et al*, 1995; Cook *et al*, 2007). Significantly decreased risks were observed in three of the four hospital-based case–control studies (Rosenberg, 1995; Harris *et al*, 2002; Moysich *et al*, 2002), although all were based on self-reported drug use after diagnosis of cancer, raising the possibility of recall bias. Of the remaining studies in which drug details were obtained before diagnosis, only three found significant inverse associations, two (but with overlapping data) with regular aspirin use (Schreinemachers and Everson, 1994; Ratnasinghe *et al*, 2004) and a third with other NSAID use (Hernández-Diaz and Rodriguez, 2007). The remaining studies, including the three low-dose aspirin randomised trials (Peto *et al*, 1988; Lee *et al*, 1995; Cook *et al*, 2007) and a Danish cohort study that we previously reported (Skriver *et al*, 2005), revealed a modest, nonsignificantly decreased risk, no appreciable effect or an increased risk, respectively. The observational studies relied either on self-reported use of NSAIDs or did not control sufficiently for smoking habits.

We examined the association between use of NSAIDs and lung cancer risk based on pooling of data from two studies. We used self-reported information on drug use as well as a continuously updated prescription database covering all pharmacies in Denmark since 1995 (Gaist *et al*, 1997), supplemented by self-reported smoking habits.

## MATERIALS AND METHODS

### Northern Jutland case–control study

For this study, we recruited patients who had been referred to one of four regional pulmonary centres in the northern part of Jutland during 2002–2005 for a broncho-mediastinoscopic examination because of an abnormal or suspect finding on a chest radiograph. We invited a total of 546 persons, of whom 416 (76%) agreed to participate. On the date of arrival of the patient at the outpatient clinic, defined as the index date, the study nurse conducted a structured face-to-face interview before a broncho-mediastinoscopy was undertaken. In addition to personal data, which included the personal identification number, the patient was asked about use of NSAIDs and acetaminophen, details of current and past smoking habits and occupational training. Of the 416 patients recruited, 165 (99 men and 66 women) were subsequently diagnosed with a biopsy-confirmed lung cancer; the remaining 251 patients (60%) were considered to have benign lung disease or an inert lung lesion and were accordingly excluded from study. We excluded a further 20 of the 165 patients with lung cancer who were listed in the Danish National Cancer Registry with a previous malignancy and one who was under the age of 40 years at the time of diagnosis, leaving 144 patients for the combined analysis. We selected 1071 control persons from the general population of northern Jutland, frequency matched on sex and age on the index date to the lung cancer patients. Of these, 449 (42%) agreed to participate and completed the same face-to-face interview as that used for the cases, but in their homes. Linkage to the files of the Cancer Registry showed that 21 of these persons had had a cancer before the index date and they were excluded, leaving 428 controls for analysis.

### Nested case–control study

This study is nested within the Danish Diet, Cancer and Health prospective cohort study described in detail elsewhere (Tjønneland *et al*, 1991, 2004). It was established by the Danish Cancer Society in 1993–1997, when 160 725 persons (80 996 men and 79 729 women) aged 50–64 years, resident in Copenhagen or Aarhus (the two largest cities in Denmark) and born in Denmark, were invited to participate; of these, 57 052 (35.5%) agreed to participate. A mailed questionnaire requested detailed information on current use of analgesics, current and past smoking habits and occupational training. Record linkage to the files of the Danish National Cancer Registry showed that 545 participants had been diagnosed with cancer before enrolment; these were excluded. Of the remaining 56 511, 429 (228 men and 201 women), diagnosed with lung cancer between the day of enrolment and 2003, formed the case group. For each case, we used random incidence density sampling to select one control subject matched to the case on sex and age, yielding a control group of 228 men and 201 women. The date of diagnosis of cases was defined as the index date.

### Non-steroidal anti-inflammatory drugs and acetaminophen data

To assess use of aspirin, other NSAIDs and acetaminophen among cases and controls in these two studies, we combined self-reported information from the questionnaire with information from a national prescription database (Danish Registry of Medical Product Statistics) at the Danish Medicines Agency (Gaist *et al*, 1997). The questionnaire included questions on use of specified pain-relieving pills (aspirin, other NSAIDs, acetaminophen) and frequency of use. The database holds records of all drug prescriptions dispensed at all pharmacies in Denmark since 1 January 1995, including the customer's personal number, type and amount of drug prescribed according to the Anatomical Therapeutic Chemical (ATC) classification system (Capellá, 1993) and date of dispensing. Indications for use and the recommended dosing schedule are not included.

For each of the 573 (144+429) cases and the 857 (428+429) controls, we identified all prescriptions for aspirin (ATC codes: B01AC06, N02BA01, N02BA51), other NSAIDs (ATC group: M01A) and acetaminophen (N02BE01) filed between 1995 to 2005. In Denmark, NSAIDs other than aspirin are available only by prescription, except for low-dose ibuprofen (200 mg per tablet), which is available over the counter (OTC) and accounts for approximately 14% of the total NSAID use (Mellemkjær *et al*, 2002). Aspirin and acetaminophen can be obtained OTC but are often prescribed for long-term use; a substantial proportion of prescribed aspirin is low dose for secondary prevention of cardiovascular disease, generally under the control of a physician.

### Statistical analysis

We estimated lung cancer risk among subjects who had reported or were registered as having any use of NSAIDs relative to that in subjects with no such use. Self-reported use of NSAIDs and acetaminophen was defined as use approximately 1–5 years prior to the index date in the northern Jutland case–control study and as intake during the year before the base line interview in the nested case–control study. This implies that slightly different windows for self-reported use were, that is, 2002–2005 in the Northern Jutland case–control study and 1993–1997 in the nested case–control study. Prescribed use in both studies was defined as any use of NSAIDs or acetaminophen 1 year or more before the index date. A lag time of 12 months from the index date was to avoid registrations of mild analgesics given to treat early symptoms of lung cancer. As the prospective Diet, Cancer and Health cohort study was initiated in December 1993 and the prescription database on 1 January 1995, no data were available on prescribed NSAIDs for lung cancer cases diagnosed between these dates; accordingly, they were excluded from analyses based on personal prescriptions. Associations were expressed as odds ratios (ORs), assessed by logistic regression in unmatched analyses, in which the matching criteria (year of birth, sex and study) were included as covariates with 95% confidence intervals (95% CI). Non-steroidal anti-inflammatory drug-specific risk estimates were adjusted for smoking habits, occupational training (unskilled worker; skilled worker; bachelor; a master's degree or higher) and concomitant use of acetaminophen. These analyses were conducted on the combined study data as well as separately on the two contributing studies. Risk associated with self-reported use was estimated separately by frequency (never, ever, monthly, weekly, daily) and by prescribed use, being the average number of prescriptions per year in the 1–3 years before the index date (0.5–2, 2–3, ⩾4), the lowest being 0.5, equivalent to at least one in a 2-year period. The statistical analyses were performed in SAS 9.1.

### Meta-analysis of previously published data

We combined information from all published studies of lung cancer in relation to aspirin and non-aspirin (or unspecified) NSAID use providing ORs, standardised mortality ratios, or other measures of risk in users relative to non-users. Where relative risks were presented only among subsets of users and not overall, we utilised the data for the consumers with the heaviest intake. Since multiple types of study design were used, for the meta-analysis, we abstracted or computed the observed and expected number of lung cancer cases among NSAID users for each study and summed them across studies to compute a summary meta-analytic relative risk measure for users of aspirin and other NSAIDs with 95% CI computed under Poisson assumptions for the numbers observed among NSAID users.

## RESULTS

The characteristics of the 573 lung cancer cases and the 857 sex- and age-matched controls are shown in Table 1; they comprised more men (55%) than women. The average age at diagnosis was 64 years. The cases comprised 26% adeno-, 24% squamous cell, 22% small-cell and 10% non-small-cell carcinomas; 6% were of other specified histological types and 3% lacked microscopic confirmation. Table 2 shows the relative risks for lung cancer associated with smoking habits and occupational training. Current smokers who had an estimated cumulative tobacco consumption of 1–14, 15–29 and 30 or more pack-years had highly significant 5-fold, 12-fold and 29-fold increased risks, respectively, compared to study subjects who had never smoked. After adjustment for smoking habits, skilled and unskilled workers had significant 2.1- and 1.7-fold higher risks, respectively, than those with higher education.

Use of acetaminophen at any time was associated with a modest but significantly increased relative risk of 1.34 (95% CI, 1.07–1.68), which, however, was reduced to a nonsignificantly increased risk of 1.11 after adjustment for smoking habits and occupational training (data not shown). Table 3 also shows that 362 (63%) of the 573 cases and 584 (68%) of the 857 controls had ever used NSAIDs; this was associated with a nonsignificant 14% decrease in risk after adjustment for age, sex, sub-study, smoking habits, length of education and use of acetaminophen (OR, 0.86). There was no clear variation in risk estimates for aspirin only, other NSAIDs only or mixed use, although risk reduction with aspirin only was slightly greater.

For self-reported use of all types of NSAIDs combined, we observed little differences in relative risks for monthly *vs* weekly *vs* daily use (Table 3). The adjusted relative risk for daily users was 0.77 (95% CI, 0.50–1.18). The trend analysis on the basis of all NSAID prescriptions dispensed at pharmacies since 1995 showed a reduced risk with increasing frequency of prescriptions. In the fully adjusted model, study subjects who received the highest number of prescriptions per year, that is at least four, had the lowest risk estimate (OR, 0.49; 95% CI, 0.28–0.84) (Table 3) composed of separate estimates of 0.43 (0.16–1.13) in the northern Jutland case–control study and 0.53 (0.27–1.06) in the case–control study nested in the prospective Diet, Cancer and Health cohort study. For the combined study material, the decreasing trend in risk with increasing number of annual prescriptions reached statistical significance (Table 3). The protective effect of heavily prescribed use of NSAIDs was more apparent for adeno-, or 0.12 (95% CI 0.03–0.51) associated with at least four prescriptions per year, than for squamous cell (RR 0.49; 95% CI 0.13–1.90) or small-cell and large-cell carcinomas combined (0.72; 0.17–2.98), although these estimates were based on small numbers (data not shown).

Table 4 shows that trends of decreasing risk of lung cancer with rising number of prescriptions were apparent for both aspirin and other NSAIDs, being slightly stronger for the latter. As previously indicated, the prescribed aspirin users were mainly users of low-dose aspirin.

Table 5 summarises results from our meta-analysis of previous investigations and shows that while case–control studies and trials reported lower risks among NSAID users, there was much less consistency among the 14 cohort studies. Across all studies, there was heterogeneity of findings, with the relative risks ranging from 0.3 to 1.4. When reported data were weighted by the expected numbers of lung cancers among NSAID users, the summary relative risk among aspirin users was 0.99 (95% CI, 0.95–1.03; *P*=0.62); for non-aspirin (or unspecified) NSAIDs, it was 0.92 (95% CI, 0.88–0.95; *P*<0.001) and for aspirin or non-aspirin NSAIDs, 0.95 (95% CI, 0.93–0.98; *P*<0.001). Adding our own results to the meta-analysis results in slightly lower overall risk estimates among NSAID users.

## DISCUSSION

In this study of 573 patients with clinically and histologically verified lung cancer and 857 population controls, we observed an overall 14% non-statistically significant decrease in risk associated with any use of aspirin and other NSAIDs after adjustment for smoking habits, length of education and concomitant use of acetaminophen. In an analysis according to self-reported frequency of use, the risk reduction increased to 23%, although still nonsignificant, among self-described daily users of NSAIDs. Using objective pharmacy records, we observed a significant trend of decreasing risk with increasing numbers of prescriptions, with a 50% reduction those among those with at least four NSAID prescriptions per year 1–3 years before the index date.

Despite the relative consistency of experimental data on COX-2 tissue expression and lung cancer risk and the inhibiting potential of NSAID treatment (Castonguay *et al*, 1998; Hosomi *et al*, 2000; Thun *et al*, 2002), the overall epidemiological evidence for a protective effect of NSAIDs on lung cancer is weak. Our meta-analysis showed a significantly lowered risk, but only by 5%. Several of the studies were limited by small sample size with imprecise risk estimates, self-reported NSAID use, possibly biased selection of controls and/or lack of information on smoking habits.

We addressed several of the above limitations. Its setting with a national health service largely removed referral and diagnostic biases. The availability of continuously updated information on NSAID use from all pharmacies in Denmark, in addition to self-reported information, helped us to minimise differential recall and error in exposure assessment. This approach allowed establishment of mutually exclusive exposure categories, including a reference group of non-users of both prescription and OTC NSAIDs. Recruitment of cases from highly specialised treatment centres and the Danish Cancer Registry ensured accurate ascertainment with minimal misclassification, and the availability of detailed information on smoking habits, length of education and use of acetaminophen reduced the risk for confounding.

Using self-reported information, use of NSAIDs was associated with a modest, non-significant reduction in risk similar for the three frequency categories. A comparison of self-reported use and prescription records among participants in the Diet, Cancer and Health cohort revealed that only 28% of individuals who reported daily use of aspirin during the preceding year were so recorded in the prescription database indicating frequent OTC purchases. Conversely, of individuals filling two or more prescriptions for aspirin within 1 year prior to enrolment, 98% reported its use in the questionnaire at enrolment; the corresponding figures for non-aspirin NSAIDs were 78 and 96%, indicating some misclassification of self-reported data. The use of self-reported data *vs* prescription based records may explain the somewhat different results of the risk analyses. Although the self-reports ascertain OTC use not obtainable in the prescription register, we tend to place more confidence in the national database, where possible recall bias is eliminated.

A limitation is that duration of prescribed NSAID use is unknown, as the nationwide prescription database was first started in 1995, preventing analyses by cumulative use, and our use of a yearly number of prescriptions issued during a relatively recent period might not be representative during the aetiologically relevant periods. We had no data on compliance with the prescriptions for NSAIDs, but the fact that the drugs were actually dispensed at pharmacies and paid in part by the customers and often refilled suggests that they were also consumed. Any non-compliance would bias the risk estimates towards the null.

Our study, which overcomes some of the limitations that may have hindered the detection of a beneficial effect of NSAIDs in previous studies, provides some support for the hypothesis that regular use of NSAIDs protects against lung cancer. The issue remains uncertain, however, and more research using detailed data on NSAID use and smoking is needed.

## Figures and Tables

**Table 1:** Descriptive characteristics of 1430 study subjects in two case–control studies of lung cancer and use of NSAIDs

	**Northern Jutland case–control study**				**Case–control study nested in diet and cancer cohort study**				**Combined study**			
**Characteristic**	**Cases**	**%**	**Controls**	**%**	**Cases**	**%**	**Controls**	**%**	**Cases**	**%**	**Controls**	**%**
*Both sexes*	144	100	428	100	429	100	429	100	573	100	857	100
Men	87	60	245	57	228	53	228	53	315	55	473	55
Women	57	40	183	43	201	47	201	47	258	45	384	45
												
*Year of birth*												
<1930	25	17	69	16	6	1	0	0	31	5	69	8
1930–1944	89	62	250	58	407	95	413	96	496	87	663	77
⩾1945	30	21	109	26	16	4	16	4	46	8	125	15
												
*Age at diagnosis*^a^ *(years)*												
<60	34	24	123	29	130	30	131	31	164	29	254	3
60–69	51	35	179	42	271	63	271	63	322	56	450	53
⩾70	59	41	126	29	28	7	27	6	87	15	153	18
												
Mean age (s.d.) (years)	67	(9)	65	(9)	63	(5)	63	(5)	64	(6)	64	(7)
												
*Year of diagnosis*												
1994–96	0	0	—	—	40	9	—	—	40	7	—	—
1997–99	0	0	—	—	146	34	—	—	146	25	—	—
2000–02	0	0	—	—	204	48	—	—	204	36	—	—
2003–05	144	100	—	—	39	9	—	—	183	32	—	—
												
Year of interview (range)	2002–2005				1993–1997				1993–2005			

a Age at diagnosis of lung cancer for cases and age at index date for controls.

**Table 2:** ORs for lung cancer by smoking habit and length of education, with associated 95% CIs

	**Cases/controls**	**Lung cancer**			
**Risk factor**	**573/857**	**OR^a^**	**95% CI**	**OR^b^**	**95% CI**
*Smoking habit*					
Never	23/284	1	Reference	—	—
					
*Former*					
>10 years since quitting	51/243	2.84	1.62–4.97	—	—
⩽10 years since quitting	52/58	16.0	8.22–31.1	—	—
					
*Current*					
1–14 pack-years	15/36	4.76	2.21–10.3	—	—
15–29 pack-years	74/77	12.4	7.19–21.5	—	—
⩾30 pack-years	353/149	28.9	17.8–47.1	—	—
					
*Age at start (years)*					
>20	51/89	1	Reference	1	Reference
15–20	342/359	1.70	1.14–2.52	1.61	1.04–2.50
<15	157/124	2.82	1.74–4.56	2.53	1.45–4.41
Missing information	5/10				
					
*Occupational training*					
Master's degree or higher	73/137	1	Reference	1	Reference
Bachelor's degree	149/250	1.27	0.87–1.85	1.10	0.69–1.76
Skilled worker	170/272	2.25	1.49–3.41	2.07	1.27–3.38
Unskilled worker	173/189	2.91	1.91–4.44	1.68	1.02–2.76
Missing information	8/9				

CI=confidence interval; OR, odds ratio.

a Adjusted for age, sex and study.

b Adjusted for age, sex, study and smoking habit.

**Table 3:** ORs for lung cancer and 95% CIs by pattern of use of NSAIDs

	**Cases/controls**	**Full study**			**Northern Jutland**	**Diet and cancer cohort**
**Use of NSAIDS or acetaminophen**	**573/857**	**OR^a^**	**OR^b^**	**95% CI**	**OR^b^**	**OR^b^**
*NSAIDs*						
Never^c^	211/273	1	1	Reference	1	1
Any	362/584	0.92	0.86	0.65–1.14	0.78	0.91
Aspirin only	71/117	0.76	0.75	0.49–1.14	0.92	0.64
Other NSAIDs only	156/273	0.90	0.87	0.62–1.23	0.63	1.07
Mixed	135/194	1.09	0.94	0.65–1.35	0.98	0.97
						
*Self-reported*						
Monthly	63/100	0.78	0.82	0.52–1.27	0.85	0.85
Weekly	47/64	0.82	0.79	0.46–1.34	1.16	0.79
Daily	86/161	1.12	0.77	0.50–1.18	0.73	0.84
						
*Prescriptions/year* d						
0.5–2	122/188	0.98	0.90	0.62–1.31	0.59	1.10
2–3	54/86	0.95	0.81	0.50–1.32	0.83	0.67
⩾4	33/79	0.66	0.49	0.28–0.84	0.43	0.53
						
Test for trend (*P*)		0.30	0.02		0.07	0.32

CI=confidence interval; OR, odds ratio.

Adjusted estimates are given for the combined study as well as for the Northern Jutland and Diet and Cancer cohort sub-studies.

a Adjusted for age, sex and study.

b Adjusted for age, sex, study, smoking habit, length of education and use of acetaminophen.

c No self-reported use or prescriptions >1 year before index date.

d Exposure 1–3 years before index date.

**Table 4:** ORs for lung cancer and 95% CIs by prescribed use of aspirin and other NSAIDs

	**Aspirin^b^**			**Other NSAIDs**		
**Prescriptions/year^a^**	**Cases/controls**	**OR^c^**	**95% CI**	**Cases/controls**	**OR^c^**	**95% CI**
Never	211/273	1	—	211/273	1	—
0.5–2	34/48	0.83	0.45–1.53	112/190	0.84	0.57–1.22
2–3	36/69	0.62	0.36–1.06	26/43	0.77	0.40–1.47
⩾4	14/22	0.74	0.32–1.71	13/36	0.41	0.18–0.93
						
Test for trend (*P*)		0.09		0.06		

CI=confidence interval; OR, odds ratio.

a Exposure 1–3 years before index date.

b Mainly low-dose aspirin for secondary prevention of cardiovascular disease.

c Fully adjusted model.

**Table 5:** Meta-analysis of studies of lung cancer in relation to NSAID use

**Study type**	**First author (year)**	**Location**	**Sex**	**NSAID evaluated**	**Number of lung cancer cases in users**	**RR**	**95% CI**
Case–control	Rosenberg (1995)	US	M+F	naNSAIDs	72	0.8	0.6–1.2
	Harris *et al* (2002)	US	M+F	NSAIDs	55	0.32	0.23–0.44
	Moysich *et al* (2002)	US	M+F	ASA	121	0.57	0.41–0.78
	Muscat *et al* (2003)	US	M+F	NSAIDs	174	0.68	0.53–0.89
							
Cohort	Paganini-Hill *et al* (1989)	US	M	ASA	15	1.35	NS
		US	F	ASA	2	0.29	NS
	Thun *et al* (1993)	US	M	ASA	210	1.11	0.98–1.25
		US	F	ASA	157	1.07	0.88–1.30
	Schreinemachers and Everson (1994)	US	M	ASA	40	0.55	0.38–0.81
		US	F	ASA	32	1.40	0.74–2.66
	Akhmedkhanov *et al* (2002)	US	F	ASA	15	0.66	0.34–1.28
	Friis *et al* (2003)	Denmark	M	ASA	173	1.0	0.8–1.1
		Denmark	F	ASA	101	1.3	1.0–1.5
	Sørensen *et al* (2003)	Denmark	M+F	naNSAIDs	692	1.1	1.0–1.2
	Holick *et al* (2003)	US	M	ASA	257	1.13	0.89–1.43
	Ratnasinghe *et al* (2004)	US	M+F	ASA	232	0.81	0.62–1.07
	Skriver *et al* (2005)	Denmark	M+F	naNSAIDs	226	1.39	1.23–1.57
	Hayes *et al* (2006)	US	F	ASA	109	1.08	0.81–1.45
		US	F	naNSAIDs	61	1.10	0.80–1.51
	Jacobs *et al* (2007)	US	M+F	ASA	85	0.98	0.76–1.25
	Wall *et al* (2007)	US	M+F	naNSAIDs	280	0.96	0.82–1.11
	Langman *et al* (2000)	UK	M+F	NSAIDs	172	0.84	0.69–1.02
	Hernández-Diaz and Rodriguez (2007)	UK	M+F	ASA	958	1.12	0.99–1.28
			M+F	naNSAIDs	938	0.89	0.79–1.00
							
Trial	Peto *et al* (1988)	UK	M	ASA	14	0.64	NS
	Lee *et al* (1995)	US	M	ASA	64	0.88	0.62–1.25
	Cook *et al* (2007)	US	F	ASA	90	0.78	0.59–1.03
							
All studies				ASA	2675	0.99	0.95–1.03
				NSAID	2670	0.92	0.88–0.95
				ASA/NSAID	5345	0.95	0.93–0.98

naNSAID=non-aspirin (or unspecified) NSAID; ASA=aspirin; NS=not specified.
